# Extracellular Vesicles as Master Regulators of Immune Modulation in Multiple Myeloma

**DOI:** 10.3390/ijms27146276

**Published:** 2026-07-14

**Authors:** Marzia Pucci, Elisa Costanzo, Martina Marfia, Gregorio Seidita, Simona Fontana, Chiara Corrado, Riccardo Alessandro

**Affiliations:** 1 Department of Biomedicine, Neuroscience and Advanced Diagnostics, University of Palermo, Via Divisi 83, 90133 Palermo, Italy; marzia.pucci@unipa.it (M.P.); elisa.costanzo01@unipa.it (E.C.); martina.marfia01@unipa.it (M.M.); gregorio.seidita@unipa.it (G.S.); simona.fontana@unipa.it (S.F.); riccardo.alessandro@unipa.it (R.A.); 2 Institute for Biomedical Research and Innovation (IRIB), National Research Council (CNR), Via Ugo La Malfa 153, 90146 Palermo, Italy

**Keywords:** multiple myeloma, immunosuppression, extracellular vesicles (EVs)

## Abstract

Multiple myeloma (MM) is a genetically and clinically heterogeneous plasma cell malignancy characterised by clonal expansion of differentiated B cells within the bone marrow (BM). Patients start with monoclonal gammopathy of undetermined significance (MGUS) and progress to an intermediate stage called smouldering multiple myeloma (SMM), characterised by several genetic alterations that represent the genomic backbone of the malignant clone. Immune checkpoint pathways play a central role in shaping an immunosuppressive BM niche, contributing to T-cell dysfunction, immune evasion, and therapeutic resistance. Key inhibitory receptors such as PD-1, CTLA-4, TIM-3, LAG-3, and CD47 are frequently dysregulated, promoting T-cell exhaustion, anergy, and senescence. Emerging evidence highlights extracellular vesicles (EVs) as critical mediators of intercellular communication in MM. MM-derived EVs carry bioactive cargo, including proteins and miRNAs, that reprogram immune and stromal cells, enhancing tumour progression and immune escape. Notably, EV-associated immune checkpoint molecules contribute to the establishment of a permissive microenvironment. This review provides an integrated overview of immune checkpoint dysregulation and EV-mediated immunomodulation in MM, emphasising their role in disease pathogenesis and progression. Furthermore, we discuss the therapeutic potential of targeting immune checkpoints and exploiting EVs as novel biomarkers and drug delivery systems, highlighting their promise for improving precision medicine approaches in MM.

## 1. Background on Multiple Myeloma

Multiple myeloma (MM) is a plasma cell disorder marked by clinical heterogeneity and characterised by clonal expansion of differentiated B cells within the bone marrow (BM), thus inducing progressive lytic bone disease, renal failure, anaemia, immunodeficiency, increased susceptibility to infections and early death [1,2].

MM is a heterogeneous and genetically complex disease; the prognosis is mainly based on chromosomal abnormalities, which are present in up to 90% of patients at diagnosis [3]. Therefore, during the diagnostic process, chromosome analysis represents the gold standard for selecting an appropriate therapeutic strategy [4,5]. Unfortunately, the disease progression is a multi-step process that leads to the subclonal expansion of cells with secondary genomic defects and alterations in the BM microenvironment, culminating in tumour formation [6].

The first stage of the disease is an asymptomatic condition known as monoclonal gammopathy of undetermined significance (MGUS), characterised by early genomic events such as hyperdiploidy and immunoglobulin heavy chain (IgH) translocations, and <10% of clonal plasma cells in the BM (BMPCs). Patients then progress to an intermediate stage called smouldering multiple myeloma (SMM), requiring ≥10% of BMPCs and characterised by additional genetic alterations that precede symptomatic disease by years [5,6]. These initiating alterations form the genomic backbone of the malignant clone, which subsequently acquires further secondary genetic events, epigenetic alterations, metabolic reprogramming and altered adhesion. Together, these events collectively contribute to disease progression, phenotypic heterogeneity, and the emergence of aggressive subclones that often develop therapeutic resistance [7,8].

Furthermore, the pathogenesis of MM is strictly dependent on the BM microenvironment and its effects on malignant plasma cells [9]. BMPCs interact with non-tumour cells, such as stromal and endothelial cells, osteoblasts, osteoclasts, and immune cells. These surrounding cells induce the secretion of cytokines and growth factors, thereby promoting survival and supporting ongoing proliferation, immune evasion, and MM development [10,11]. For example, Peng and colleagues demonstrated that Insulin-like Growth Factor-1 (IGF-1) promotes progression in multiple myeloma through Phosphoinositide 3 kinase-Protein Kinase B-Akt (PI3K/Akt)-mediated epithelial–mesenchymal transition [12]. Jiang et al. showed the anti-tumour effects of Raddeanin A, a natural compound extracted from Anemone raddeana Regel, on cell cycle arrest and apoptosis of MM cells through the MAPK/ERK signalling pathway [13]. Molecular studies on BM samples from patients with MGUS, SMM and MM have also highlighted immunophenotypic alterations within the tumour microenvironment, such as dysregulation of MYC signalling and Early Region 2 binding Factor (E2F) targets; furthermore, interferon release becomes predominant when the disease becomes symptomatic, which correlates with its progression [14,15]. Moreover, analysis with a multi-modal single-cell omics approach has identified, in MGUS, early dysregulation of the immune system: population shifts in CD8^+^ T-cells, macrophages polarise towards an M1 phenotype, antigen-presenting cell population decrease, as well as other changes in the tumour and in its microenvironment. These events increase in SMM and MM, correlating with adverse clinical outcomes [14,16].

From an epidemiological perspective, the incidence rate has risen, and MM is now the second most common blood cancer worldwide. MM constitutes about 1.8% of all newly diagnosed cancer cases and 2% of deaths related to cancer in 2024 in the USA, with a median survival rate of 6 years [17,18,19]. MM prognosis criteria have changed over time due to the evolution of the pathology, increased heterogeneity, and mainly advances in the overall understanding of the disease. Clinically, MM patients present with a broad spectrum of manifestations related to the CRAB features—hyperCalcaemia, Renal impairment, Anaemia, and Bone lesions—which directly result from plasma cell infiltration. Diagnosis is based on serum protein electrophoresis, the measurement of kappa and lambda immunoglobulin light chains through serum free light chain (FLC) assays, and fluorescence in situ hybridization (FISH) to identify cytogenetic abnormalities that impact risk stratification. Furthermore, diagnosis can be based on the identification of bone lesions or extramedullary diseases by whole-body low-dose computed tomography (WBLDCT) and positron emission tomography (PET/CT) [20].

Several MM classification schemes are defined: the International Staging System (ISS) was the first to be introduced and was based on available prognostic biomarkers (e.g., serum beta-2 microglobulin (B2M), and albumin) [21]. Subsequently, it was updated by the Revised International Staging System (R-ISS) which included, in addition to prognostic biomarkers, chromosomal defects, identified through genomic techniques [22]. The International Myeloma Working Group (IMWG) updated the ISS and divides patients into four groups with different risks of progression based on different parameters such as BMPCs ≥ 20%, specific biomarkers and the presence of cytogenetic abnormalities [23]. Recently, the R2-ISS (an updated version of the R-ISS) and the Mayo Additive Staging System (MASS) were introduced, adding point scores for specific chromosomal abnormalities and stratifying patients according to risk status [17,22,24,25,26,27,28].

Currently, several clinical trials are ongoing in patients with high-risk SMM, such as the combination of lenalidomide with dexamethasone [29,30] or the triplet regimens comprising lenalidomide and dexamethasone, plus elotuzumab [31], ixazomib [32], or carfilzomib [33]. Another promising approach is a BCMA (B Cell Maturation Antigen)-directed CAR-T (Chimeric Antigen Receptor T-cell) therapy such as anitocabtagene autoleucel (anito-cel), an autologous D-Domain BCMA-directed chimeric antigen receptor (CAR) T-cell therapy that has shown 100% overall response rate (ORR), and 24-month progression-free survival (PFS) rate of 56% [34].

In recent years, the therapeutic landscape of MM has been profoundly transformed by the introduction of monoclonal antibodies. Despite the remarkable success of immune checkpoint inhibitors (ICIs) in a wide range of solid tumours, their application in MM has been considerably less effective and remains controversial. Early clinical studies investigating PD-1 blockade with agents such as nivolumab and pembrolizumab as monotherapy in relapsed/refractory MM reported minimal clinical activity, with low objective response rates and no durable tumour regressions [35].

However, Daratumumab has emerged as a breakthrough agent. Notably, its activity as a single agent, including in early disease stages such as intermediate- and high-risk smouldering MM, has demonstrated significant clinical benefit and a favourable safety profile, as shown in the final analysis of the CENTAURUS study [36]. These findings have supported its incorporation into first-line treatment strategies, underscoring the growing relevance of immune-based approaches in MM.

Despite substantial therapeutic advances in recent decades, there is an increasing trend in the global incidence of MM [37]. This is due to the persistence of measurable residual disease and the development of treatment-resistant subclones driven by ongoing clonal evolution [9,38]. A deeper understanding of the genomic and epigenetic mechanisms and of the potential role of tumour microenvironmental mechanisms governing MM initiation and progression continues to refine risk stratification and guide therapeutic innovations and precision/personalised medicine approaches, thereby improving survival and quality of life for patients with MM and its precursors [3].

## 2. Multiple Myeloma and Immune Checkpoints

Accumulating evidence indicates that the BM tumour microenvironment plays a critical role in disease progression [39,40,41]. Distinct alterations in immune checkpoint pathways within the BM microenvironment contribute to disease evolution and treatment response across the spectrum from premalignant stages (MGUS and SMM) to active MM. These alterations promote the development of an immunosuppressive niche that supports tumour survival and immune evasion [42,43]. Early and recurrent immune changes include dysfunctional dendritic cells with reduced HLA-DR expression, expansion of myeloid-derived suppressor cells, and alterations in natural killer (NK)- and T-cell compartments that impair antigen presentation and cytotoxic immune responses [44,45]. Several studies reported a progressive loss of cytotoxic CD8^+^ T-cells, depletion of progenitor and transitional B cells, and enrichment of monocytic and neutrophil populations during disease progression [46,47,48]. Inhibitory immune checkpoint proteins such as Programmed Death-1 (PD-1), Cytotoxic T-Lymphocyte Antigen 4 (CTLA-4), Lymphocyte-activation gene 3 (LAG-3), T-cell immunoglobulin and mucin domain-containing three (TIM-3), and Cluster of Differentiation 47 (CD47) play key roles in regulating T-cell activation and maintaining immune homeostasis in MM. However, their overexpression in the tumour microenvironment contributes to T-cell exhaustion and impaired anti-tumour immunity [37,44,46,49]. In light of the above, it is necessary to contribute to a better understanding of immune dysregulation in MM to highlight potential new targets for immunotherapeutic strategies as well as to identify prognostic and predictive biomarkers for the most appropriate management strategy [50,51].

### 2.1. T-Cell Dysfunction in Multiple Myeloma: T-Cell Anergy, Exhaustion and Senescence

In MM, immune dysregulation plays a central role in disease onset, progression, relapse, and resistance to therapy. T-cell activation is initiated by stimuli such as infection, inflammation, or malignancy and begins with antigen-presenting cells (APCs) displaying pathogenic antigens bound to major histocompatibility complex (MHC) molecules. These complexes are recognised by T-cell receptors (TCRs), providing the first activation signal. However, full T-cell activation and proliferation require a second co-stimulatory signal, primarily mediated by the interaction between CD28 on T-cells and its ligands CD80 and CD86 on APCs. Conversely, T-cells also express inhibitory receptors including PD-1, LAG-3, and TIM-3 which, upon ligand binding, suppress T-cell activation. In the absence of adequate CD28-mediated co-stimulation, T-cells enter a state of anergy, characterised by reduced responsiveness and impaired interleukin-2 (IL-2) production [52]. Anergy typically arises from insufficient co-stimulatory signals and/or excessive co-inhibitory signalling, leading to functional impairment of T-cells. These regulatory mechanisms are largely governed by interactions between CD28 family receptors and ligands of the B7 family such as CD80, CD86, Programmed Death-Ligand 1 and 2 (PD-L1 and PD-L2) [52]. In MM, several studies have shown that a substantial proportion of T-cells in the bone marrow (BM) are CD28^−^ in both healthy donors (HD) and patients, consistent with the presence of CD28-negative T-cell subsets under physiological conditions [53]. This feature, which is important for effective immune surveillance, suggests that T-cells in MM may adopt an anergic state [53,54]. Chronic antigenic stimulation, as seen in persistent infections or malignancies, can also drive T-cells into a state of exhaustion. T-cell exhaustion is characterised by progressive loss of effector function, reduced cytokine production, and sustained overexpression of inhibitory receptors [55]. This dysfunctional state, commonly observed in cancer, limits the ability of T-cells to control tumour growth. Among inhibitory immune checkpoint molecules, PD-1 has been the most extensively studied and was found to be highly expressed on exhausted T-cells in MM [53,56,57,58]. In addition to PD-1, other inhibitory receptors including V-domain Ig suppressor of T-cell activation (VISTA) and LAG-3 are overexpressed on exhausted CD8+ T-cells in the MM BM microenvironment and may correlate with disease relapse and poor post-transplant outcomes [53,59,60].

T-cell senescence represents another mechanism contributing to immune dysfunction in MM and has distinct features. For example, senescent T-cell subsets appear to develop independently of telomere shortening. Moreover, classical senescence-associated pathways such as p38 MAPK, p16, and p21 do not appear to be upregulated in MM. Instead, increased SMAD signalling, linked to T-cell inactivation, may play a key role in inducing cell cycle arrest [54]. The phenotypic characterisation of senescent T-cells was initially defined by a CD57^+^CD28^−^ phenotype; however, senescent T-cells are now known to express additional markers, including CD160, KLRG1, and TIM-3 [53,54]. Moreover, TIGIT and Helios have also been recently identified as key markers [61,62].

### 2.2. Inappropriate Expression of Checkpoints in Multiple Myeloma Patients

#### 2.2.1. Cytotoxic T-Lymphocyte-Associated Antigen 4 (CTLA-4), Programmed Cell Death Protein 1 (PD-1) and Its Ligands (PD-L1/PD-L2)

CTLA-4 is an inhibitory immune checkpoint that is highly expressed on activated T-cells and regulatory T-cells (Tregs) [63]. It competes with CD28 for binding to the ligands B7-1 and B7-2 on APCs, but with significantly greater affinity. As a result, CTLA-4 effectively dampens CD28-mediated T-cell activation. Another inhibitory immune checkpoint pathway involves PD-1 and its ligands PD-L1 and PD-L2. PD-1 is a type I transmembrane protein belonging to the CD28 receptor family and is expressed on various activated immune cells, including CD4^+^ and CD8^+^ T lymphocytes, B cells, NK T-cells, and APCs [64,65]. In contrast to CTLA-4, which primarily regulates early T-cell activation within lymphoid organs, the PD-1 pathway mainly controls T-cell activity during later and sustained immune responses. When PD-1 binds to its ligand, T-cell function is transiently suppressed. Consequently, T-cells enter the state known as “exhaustion” [63,66,67,68]. Evidence from the literature indicates that dysregulated expression of PD-1 and CTLA-4 on CD4^+^ T-cells in patients with MM is associated with poor clinical outcomes and disease status. Kulikowska de Nałęcz et al. reported that newly diagnosed MM patients exhibit, in their CD4^+^ T-cells, reduced levels of PD-1, CTLA-4, and of the activation marker CD69, whereas relapsed/refractory patients (RRMM) display restored expression and reactivity [69]. While, in MM, increased PD-1 expression has been reported on CD4^+^ T-cells, findings for CD8^+^ T-cells are less consistent [70]. However, in advanced stages of the disease, both CD4^+^ and CD8^+^ T-cells often show upregulated PD-1 expression compared with HD [71].

PD-L1 has a central role in regulating the immune system through its binding to PD-1. In healthy cells, it prevents autoimmune responses; in tumours, by contrast, high levels of PD-L1 are able to mask immune system activity.

Several studies have demonstrated that PD-L1 is expressed on malignant plasma cells (PCs), while it is largely absent on normal PCs from HD [57,70]. Moreover, PD-L1 expression appears to be higher in MM and SMM compared with MGUS [57,72]. Myeloid-derived suppressor cells (MDSCs), a heterogeneous population of immature myeloid cells with potent immunosuppressive activity, are also expanded in MM and are implicated in disease progression [73]. MDSCs from patients with relapsed or refractory disease exhibit higher PD-L1 expression compared to those from newly diagnosed patients [70].

#### 2.2.2. T-Cell Immunoglobulin and Mucin-Domain Containing-3 (TIM-3)

TIM-3 is a critical negative regulator of immune responses and plays a central role in shaping the tumour immune microenvironment. In patients with MM, TIM-3 is expressed on lymphocytes and contributes to tumour immune evasion [74,75,76,77,78,79,80,81]. Liu, Z et al. examined TIM-3 expression in BM-derived myeloma cells (CD38^+^CD138^+^) from MM patients and healthy donors, linking higher levels to reduced haemoglobin and red blood cell counts, suggesting an association with disease severity and anaemia [82].

Valuskova et al. investigated immune checkpoint dynamics in the BM microenvironment, showing upregulation of the inhibitory molecule PD-1 on CD4+ and CD8+ T-cells and expression of inhibitory receptors (TIM-3, TIGIT, CTLA-4, BTLA, LAG-3) that promote immune dysfunction and reinforce the immunosuppressive niche [83].

#### 2.2.3. Lymphocyte-Activation Gene 3 (LAG-3)

LAG-3, expressed on a variety of immune cells, is an inhibitory immune checkpoint receptor that functions as an inhibitory transmembrane protein. LAG-3 shares considerable homology with CD4 and primarily binds to MHC class II molecules, with a higher affinity than CD4. This interaction suppresses the activation, proliferation, and effector functions of both CD4^+^ and CD8^+^ T lymphocytes [84]. During chronic antigen exposure, such as in persistent infections or malignancies, T-cells often exhibit increased LAG-3 expression along with other inhibitory receptors, contributing to T-cell dysfunction [85]. Lucas et al. [86] examined the impact of autologous stem cell transplantation (ASCT) on the immune system in MM, analysing patients before and 90 days after transplant. They found that increased LAG-3 expression on T-cells post-ASCT was strongly associated with poorer event-free survival, suggesting a higher risk of disease progression or relapse. These findings identify LAG-3 as a potential early biomarker and therapeutic target, as its blockade enhances T-cell proliferation and cytotoxicity against myeloma cells. Chen et al. reported increased PD-1 and LAG-3 expression on CD4^+^ and CD8^+^ T-cells in RRMM, which was correlated with disease severity [87].

#### 2.2.4. CD47

One mechanism by which MM cells evade the immune system is through high expression of CD47, an inhibitory immune checkpoint known as the “do not eat me” signal, which sends inhibitory signals to macrophages to prevent phagocytosis. To investigate the potential of CD47 as a therapeutic target, Kim et al. [88] analysed BM samples from myeloma patients by flow cytometry and found elevated CD47 expression in 73% of patients compared with non-malignant BM cells. In xenograft models, anti-CD47 antibodies significantly inhibited tumour growth and induced regression in 42 of 57 mice, consistent with the elimination of myeloma-initiating cells [88]. Sun et al. found that CD47 expression correlates with disease progression, increasing from normal cells to MGUS and then to MM [89]. These findings highlight CD47 as a specific marker and potential therapeutic target for MM, suggesting that checkpoint immunotherapy targeting the CD47 “do not eat me” signal is a promising new strategy for promoting macrophage-mediated killing of MM cells.

## 3. Immune-Modulatory Properties of MM-Derived Extracellular Vesicles

Extracellular vesicles (EVs) are nanosized, membrane-enclosed particles released by nearly all cell types. Based on size, EVs are broadly classified into large EVs (>200 nm in diameter) and small EVs (<200 nm in diameter) [90]. They carry a wide range of bioactive molecules, including proteins, lipids, and nucleic acids, which can be specifically packaged by donor cells and delivered to recipient T-cells. Under physiological conditions, this process mediates essential intercellular communication within the organism. In contrast, in the context of cancer, EV-mediated communication contributes to tumour initiation, growth, and metastasis [91].

**Figure 1:**
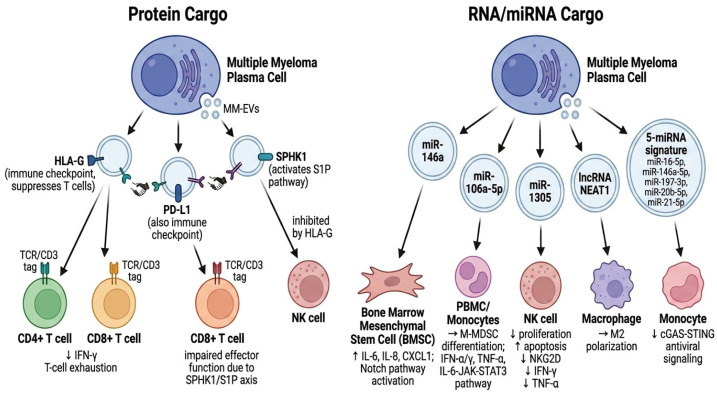
Schematic overview of the molecular cargo of MM-EVs and its role as a master regulator of immune modulation in MM. The figure was generated using the AI-assisted design tool FigureLabs (figurelabs.ai).

A growing body of evidence demonstrates that EVs play a central role in MM progression, contributing to several key pathological mechanisms, including angiogenesis [92,93,94], bone remodelling [95,96,97,98], drug resistance [99,100], and, of particular relevance to MM pathogenesis, immune dysregulation [101,102,103]. MM cells rely on dynamic interactions with the BM niche to support their survival and proliferation, and MM-derived EVs (MM-EVs) play a critical role by transferring bioactive cargo that functionally reprogrammes both immune cells and BM mesenchymal stromal cells (BMSCs) [104]. The functional effects of MM-EVs are driven by their specific molecular cargo, which reflects the physiological and pathological state of the cell of origin. The following sections will provide a detailed overview of the molecular cargo of MM-EVs and its role as a master regulator of immune modulation in MM, as illustrated in Figure 1.

### 3.1. MM-EV Protein Cargo

MM-EVs carry a rich protein cargo that contributes to immune evasion and tumour progression. These proteins can engage receptors on immune cells, thereby modulating their activation and function. Mechanistically, EVs retain membrane components from their donor cells, effectively transferring these regulatory signals to recipient T-cells. This principle is exemplified by the presence of key immune checkpoint regulators on MM-EVs. Across multiple cancer types, EVs act as vehicles for checkpoint signalling, extending tumour-mediated immunosuppression beyond direct T-cell-to-cell interactions.

A key example of an immune-suppressive checkpoint molecule expressed on MM-EVs is the Human Leukocyte Antigen (HLA-G). HLA-G is a non-polymorphic HLA class I molecule that exists in both membrane-bound and soluble forms and interacts with inhibitory receptors on immune cells, including T lymphocytes and NK cells [105]. HLA-G–positive EVs have been identified across multiple tumour types, where their abundance correlates with disease progression [106]. In MM, HLA-G expression has been reported in both soluble and membrane-bound forms in cell lines and primary malignant plasma cells [107,108]. Notably, Soncini et al. [109] described BM-derived EVs from MM patients expressing higher levels of HLA-G compared with HD. Importantly, these authors also demonstrated a functional role for HLA-G^+^ EVs: exposure of CD4^+^ and CD8^+^ T-cells to MM-EVs resulted in a significant reduction in Interferon-γ (IFN-γ) production. This effect was specifically mediated by HLA-G, as antibody blockade restored IFN-γ secretion, highlighting the immunosuppressive capacity of HLA-G^+^ EVs [109].

Similarly, EVs carrying the inhibitory checkpoint PD-L1 and its receptor PD-1 have been reported in multiple cancer types, including melanoma, lung cancer, breast cancer, glioblastoma, head and neck squamous cell carcinoma, and gastric cancer [110,111,112,113,114,115,116,117]. In MM, PD-L1 has been detected in EVs isolated from both MM cell lines and the BM plasma of patients, further supporting the immunoregulatory role of EV-associated checkpoints [118].

In addition to checkpoint molecules, other bioactive components of MM-EVs contribute to immune dysregulation. For instance, Hagiwara et al. [119] identified sphingosine kinase 1 (SPHK1) as an EV-enriched factor implicated in immunosuppression. SPHK1 catalyses the conversion of sphingosine into sphingosine-1-phosphate (S1P), a bioactive lipid involved in immune regulation, lymphocyte trafficking, tumour progression, and drug resistance. SPHK1 transfer via EVs to CD8^+^ T-cells activates the SPHK1/S1P signalling axis in recipient T-cells, leading to impaired effector function, including a reduced proportion of IFN-γ–producing T-cells [119].

### 3.2. MM-EV RNA/microRNA Cargo

The bioactive cargo of EVs transferred to recipient T-cells can also reprogramme their gene expression by delivering microRNA (miRNAs) and mRNAs [120], which reshape the phenotype of target T-cells [98,121].

MM-EV-associated miRNAs modulate several pathways linked to immune modulation, including tumour progression, angiogenesis, osteolytic bone disease, and immune suppression, which are key drivers of MM disease [92,98,122,123,124]. Collectively, these pathways orchestrate a profound remodelling of the BM niche by expanding immunosuppressive cellular subsets, enhancing the production and release of pro-tumourigenic inflammatory cytokines, and impairing cytotoxic lymphocyte activity. Importantly, this cytokine network operates within a highly dynamic BM microenvironment, where reciprocal interactions between tumour cells and niche components amplify cytokine release and signalling, ultimately establishing a permissive microenvironment that facilitates MM immune evasion [125,126,127].

De Veirman et al. demonstrated that MM-EVs are enriched in miR-146a, which is transferred to BMSCs and promotes BM-MSc to secrete Interleukin-6 (IL-6), IL-8 and C-X-C motif ligand 1 (CXCL1), which are known to drive MM cell growth and migration through activation of the Notch pathway [123].

In the context of immune suppression, it is well established that MDSCs expand within the BM from the earliest stages of MM, rapidly becoming a dominant immunosuppressive population that blocks T-cell activation and weakens anti-tumour immunity [128]. MM-EVs can reinforce this process by delivering miR-106a-5p and miR-146a-5p to healthy peripheral blood mononuclear cells, driving their differentiation into monocytic myeloid-derived suppressor cells (M-MDSCs). These miRNAs subsequently enhance the expression of multiple immunomodulating mediators, including genes associated with Interferon α (IFN-α) and IFN-γ responses, inflammatory signalling, tumour necrosis factor-α (TNF-α) pathways, and Interleukin-6-Janus Kinase-Signal Transducer and Activator of Transcription 3 (IL-6–JAK–STAT3) signalling, thereby strengthening an immunosuppressive microenvironment [124].

NK-cell activity is also impaired by MM-EV-mediated transfer of the long non-coding RNA (lncRNA) NEAT1. NEAT1 suppresses NK-cell proliferation, promotes apoptosis, reduces NKG2D expression, and diminishes the production of TNF-α and IFN-γ. Using NEAT1-silenced exosomes, authors demonstrated that exosomal NEAT1 regulates the EZH2/PBX1 axis to inhibit NK-cell activity, thereby promoting MM cell immune escape [129].

Beyond NK-cell suppression, MM-EVs also target other innate immune compartments. Lee et al. [130], following the culture of hypoxic MM cell lines, showed that exosome release was markedly increased compared with normoxic conditions. miRNA profiling of hypoxia-derived MM exosomes revealed that miR-1305 was significantly upregulated. When miR-1305 expression was correlated with clinical outcome, higher levels of this miRNA were associated with poorer overall survival rates in MM patients. Importantly, this miR-1305 can be transferred to macrophages via MM-EVs, driving their conversion forward a tumour-promoting M2 phenotype [130]. Innate antiviral immune responses can also be inhibited by MM-EVs. This occurs as MM patient-derived EVs transfer five miRNAs (miR-16-5p, miR-146a-5p, miR-197-3p, miR-20b-5p and miR-21-5p) to monocytes, thereby blocking the cyclic guanosine monophosphate (cGMP)–adenosine monophosphate (AMP) synthase and stimulator of interferon genes cGAS-STING signalling pathway, which is normally activated in monocytes/macrophages upon viral infection and induces type I interferon responses upon viral infection. Notably, patients with high risk scores based on this 5-miRNA signature appear to be more susceptible to viral infection than those with low-risk scores. These findings indicate that exosomal miRNAs are highly valuable biomarkers for predicting infection risk in MM patients [131].

## 4. Immune-Modulatory Effects of MM-EVs on the Tumour Microenvironment

**Figure 2:**
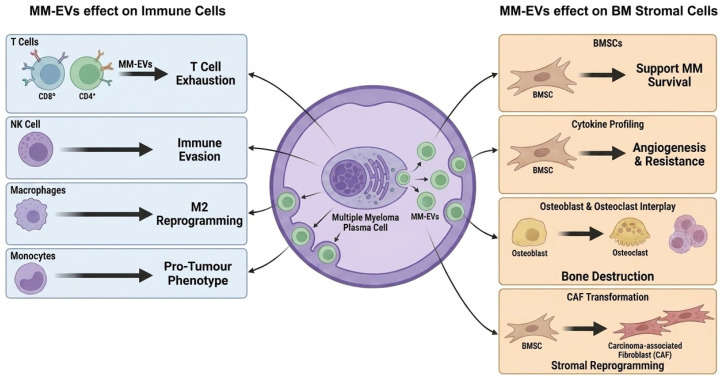
Overview of the immune-modulatory effects of MM-EVs on both immune and BM cellular compartments. The figure was generated using the AI-assisted design tool FigureLabs (figurelabs.ai).

In the light of their rich and heterogeneous molecular cargo, MM-EVs act as key mediators of immunomodulation within the BM microenvironment. They modulate both BM-resident T-cells, including stromal cells, endothelial cells, osteoblasts, and osteoclasts, and immune cells, such as T-cells, NK cells, dendritic cells, and macrophages. The following paragraphs will provide a more detailed overview of the immune-modulatory effects of MM-EVs on both immune and BM cellular compartments, which are summarised in Figure 2.

### 4.1. Immune-Modulatory Effects of MM-EVs on Immune Cells

In the context of T-cell dysfunction in MM, it has been shown that co-culture of CD8^+^ T-cells with MM-EVs, isolated either from the plasma of MM patients or from MM cell lines, leads to a significant increase in the proportion of T-cells expressing the immune inhibitory checkpoint receptors PD-1, TIGIT, and LAG-3. This phenotype is accompanied by a reduced frequency of IFN-γ-producing CD8^+^ T-cells, indicating impaired effector function and supporting the development of an exhausted T-cell state [119]. Consistent with these findings, Lopes et al. [132] demonstrated that conditioning BALB/cByJ mice by injecting MM-EVs derived from the MOPC315.BM cell line resulted in the establishment of an immunosuppressive milieu. In particular, CD4^+^ T-cells acquired a pro-tumour profile characterised by increased expression of the immune inhibitory checkpoint molecules PD-1 and CTLA-4 compared to control mice. Notably, these effects occurred in the absence of malignant plasma cells, indicating that MOPC315. BM-derived EVs alone are sufficient to suppress CD4^+^ T-cell–mediated anti-tumour immunity and promote immune evasion [132].

Within the innate immune compartment, NK cells play a central role in the immunosurveillance of MM due to their ability to produce a broad range of cytokines and chemokines, as well as to directly kill MM cells. The activating receptor Natural Killer Group 2-member D (NKG2D) is a key mediator of NK-cell-dependent recognition and elimination of MM cells [129]. NKG2D ligands (NKG2DLs) are generally considered “danger signals” that mark stressed or transformed cells for immune clearance. However, the release of NKG2DLs into the extracellular milieu, either through proteolytic shedding or via EV secretion, represents a mechanism that regulates their surface expression and can be exploited by tumour cells to evade NKG2D-mediated immunosurveillance [133]. NKG2DLs are stress-inducible molecules that in humans belong mainly to two families: the MHC class I chain-related proteins A and B (MICA/B) and the UL16-binding proteins (ULBP1–6). In MM, elevated levels of soluble MICA in patient serum have been associated with disease stage and progression [131]. Consistently, Vulpis et al. [134] reported that MM-EVs also carry substantial amounts of MICA. While acute exposure to these MICA-positive EVs can activate NK cells and enhance cytotoxic responses, chronic exposure leads to sustained downregulation of NKG2D on NK cells. This results in impaired NKG2D-dependent functions and reduced tumour cell killing, ultimately contributing to immune evasion over time [134].

Among immune cells, resident macrophages represent key immunoregulatory components that can profoundly shape the tumour microenvironment in favour of tumour progression. In this context, exposure of M0 macrophages to U266B1-derived EVs induces a time-dependent increase in M2-associated markers, including elevated expression of Arginase-1 (Arg-1) and Interleukin-10 (IL-10) at the transcriptional level. Consistently, increased surface expression of CD206 further supports the acquisition of an M2-like phenotype, indicating that MM-EVs promote macrophage reprogramming towards a tumour-supportive state and contribute to the establishment of an immunosuppressive microenvironment [135].

Moreover, Pucci et al. [118] demonstrated that MM-EVs also modulate macrophage function by upregulating PD-L1 and IL-6 expression at both mRNA and protein levels. This effect is associated with activation of the IL-6/STAT3 signalling pathway, further reinforcing an immunoregulatory and suppressive macrophage phenotype within the tumour microenvironment [118].

With regard to monocytes, MM-EVs have been reported to modulate monocyte function: uptake of EVs from the MM cell lines H929 and U266 increased IL-6 and Matrix Metalloproteinase-9 (MMP-9) secretion in a dose-dependent fashion, thus suggesting that MM-EVs can drive a pro-tumourigenic and immune-modulatory phenotype in monocytes [136].

Furthermore, Mizuhara et al. [124] demonstrated that MM-derived exosomes are internalised by PBMCs, inducing their differentiation into M-MDSCs with immunosuppressive activity. Mechanistically, this effect was mediated by the transfer of exosomal miR-106a-5p and miR-146a-5p, which activated inflammatory and immunoregulatory pathways, including IFN-α, IFN-γ, TNF-α, and IL-6/JAK/STAT3 signalling. Importantly, inhibition of these miRNAs significantly reduced M-MDSC induction, highlighting the role of MM-EVs in reshaping the immune microenvironment and promoting tumour immune evasion.

The effects of MM-EVs on different immune cell populations are summarized in Table 1.

### 4.2. Immune-Modulatory Effects of MM-EVs on BMSCs

The tumour-surrounding microenvironment plays an essential role in sustaining tumour development and shaping its immunological landscape. As MM progresses, malignant plasma cells remodel the BM niche into a pro-tumoural ecosystem by establishing a dense network of reciprocal interactions with resident and infiltrating cell populations. This bidirectional crosstalk enhances multiple tumour-supportive processes, including cell proliferation, metabolic fitness, drug resistance, and immune escape. Among the stromal components, BM-MSCs act as a key regulatory hub within the TME: they not only provide structural support, but also secrete cytokines, chemokines, and extracellular matrix components that sustain MM cell survival and foster the expansion of immunosuppressive cell subsets [137]. In addition to direct T-cell–cell interactions, recent evidence highlights an indirect interplay between BMSCs and MM cells, particularly mediated by MM-EVs, as discussed below [138,139]. Notably, several signalling pathways are highly interconnected and converge on the regulation of immune responses, further reinforcing the central role of BMSCs within the complex network of tumour–microenvironment interactions. Accumulating evidence highlights a dual role of MM-EVs: in fact, they reinforce protective pathways already activated in the tumour niche, and they can also reprogramme BMSCs, altering their phenotype and secretory profile. Reale et al. [140] demonstrated that EVs from MM cell lines and patient plasma could reshape the BM niche. These EVs enhance BMSC proliferation and migration, which in turn increases adhesion of human myeloma cell line to BMSCs. Importantly, proteomic profiling of patient-derived plasma EVs (ps-EVs) showed that MM-psEVs were enriched in key mediators of these processes compared with EVs from healthy donors [140]. This is biologically relevant as MM–BMSC adhesion is a well-established driver of tumour progression: in fact, it promotes drug resistance [141], mediates angiogenesis by upregulating vascular endothelial growth factor (VEGF) secretion, and it induces IL-6 production, which is mediated by NF-kB [142]. Through this coordinated NF-κB–driven mechanism, BMSCs reinforce MM cell proliferation, immune escape, and microenvironmental remodelling, ultimately leading to tumour formation [143].

Several other studies have highlighted that MM-EVs induce BMSCs to release essential cytokines that support MM tumour growth and tumour cell survival, such as IL-8 and IL-6. Raimondo et al. [96] found that MM-EVs boost BMSCs to release higher amounts of IL-8, which is mediated by the Epidermal Growth Factor Receptor (EGFR) pathway activation. Furthermore, exposure to conditioned medium derived from BMSCs pre-treated with MM exosomes enhanced the expression of osteoclastogenic markers, suggesting that MM-EV-educated BMSCs can promote osteoclastogenic signalling [96].

In the context of elevated IL-6 release, MM-EVs can stimulate BMSCs to produce IL-6 via the Apurinic/apyrimidinic endonuclease (Ape1)/NF-kB pathway, thereby suppressing osteoblastic differentiation [139]. This leads to expansion of the osteoclast population, which establishes a microenvironment that supports MM cell growth and survival through an IL-6-dependent mechanism [144]. Importantly, this IL-6-rich environment also contributes to stromal reprogramming. As discussed below, miRNAs carried by EVs can drive MM progression by stimulating IL-6 production in human BMSCs and their transformation into carcinoma-associated fibroblasts CAFs [120]. CAFs are known to participate in immune suppression by releasing Tumour Growth Factor-beta (TGF-*β*) and VEGF, both of which are abundant immunosuppressive factors in the MM microenvironment [145,146].

Consistently, MM-EVs derived from the OPM2 cell line have been shown to enhance MSC proliferation and to further increase IL-6 secretion, thereby inducing CAF transformation of MSCs through the regulation of two miRNAs, miR-21 and miR-146a [147].

Collectively, these findings demonstrate the pivotal role of MM-EVs in reprogramming BMSCs and reshaping the BM microenvironment into a niche that simultaneously promotes tumour growth and facilitates immune escape.

The effects of MM-EVs on BMSCs are summarized in Table 2.

## 5. Clinical Implications and Future Perspectives

### 5.1. MM-EVs as Tumour Biomarkers in Multiple Myeloma

EVs are increasingly recognised as promising tumour biomarkers because they carry disease-specific proteins, surface markers, and nucleic acids—including PD-L1, HLA-G, and specific miRNAs—that can be readily detected in biological fluids, supporting their application in disease diagnosis, prognosis, and therapeutic monitoring [148]. In MM, studies of circulating plasma- and serum-derived EVs have demonstrated their ability to distinguish patients from healthy individuals, as they harbour MM-associated molecular signatures that correlate with clinical characteristics and disease burden, highlighting their potential as liquid biopsy biomarkers [149]. Furthermore, proof-of-concept studies have identified MM-specific markers within EVs isolated from liquid biopsies, supporting their use as a non-invasive source of measurable residual disease biomarkers [150].

The intrinsic heterogeneity of EV populations and their cargo complicates the identification of robust, disease-specific signatures. However, recent advances in EV characterisation have further strengthened their clinical utility, showing that, using conventional but appropriately optimised flow cytometry platforms [151], EVs can be quantified and phenotypically characterised as measurable biological entities. Their intrinsic heterogeneity can be resolved through the analysis of surface-marker signatures using approaches such as tetraspanin profiling, multiplex bead-based assays, and immunocapture techniques, which enable the identification of distinct EV subpopulations and tumour-associated EV profiles in patient plasma [152]. In MM, these strategies facilitate the discrimination of tumour-derived EVs from those released by other cell types using MM-specific marker panels. Importantly, both tumour- and stromal-derived EV populations are increasingly recognised as biologically relevant biomarkers that reflect disease biology, the dynamic interactions within the BM microenvironment, and the evolution of MM [149].

### 5.2. Therapeutics on MM-EVs

While EV-based strategies offer promising avenues for MM management, several critical limitations must be acknowledged before clinical translation can be realistically envisaged.

Interfering with EV biogenesis, release, or uptake represents a theoretically sound strategy to disrupt tumour-stroma crosstalk [95,153]. Nevertheless, broad inhibition of EV secretion may interfere with physiological intercellular communication, potentially causing unintended toxicity [153]. Given the complex and redundant nature of EV-mediated signalling, it seems unlikely that targeting a single pathway will yield meaningful clinical benefit [154]. Moreover, pharmacological inhibitors of EV biogenesis such as GW4869 lack specificity and are unsuitable for clinical use [95]. These limitations suggest that EV-targeted therapies may be more effective as adjuvants rather than standalone treatments [154].

The use of EVs as natural nanocarriers faces substantial hurdles. Efficient and reproducible loading of therapeutic cargo remains technically challenging, with current methods often yielding low encapsulation efficiency [155,156]. Large-scale GMP-compliant production of EVs is expensive and logistically demanding [154], and biodistribution studies reveal that systemically administered EVs predominantly accumulate in the liver and spleen, with limited tumour-specific targeting unless actively engineered [157]. Additionally, the immunogenicity of EVs derived from allogeneic sources may trigger unwanted immune responses [158]. Although engineering strategies, such as surface modification with targeting ligands, may partially address these issues, their clinical feasibility and cost-effectiveness remain unproven [159].

CAR-T-cell-derived exosomes and EV-based vaccines represent promising concepts [160,161], but their translational potential is uncertain. Evidence for their efficacy in MM is largely restricted to preclinical models, with no established clinical data [162]. The immunosuppressive cargo of tumour-derived EVs poses a risk of inadvertently promoting immune evasion rather than anti-tumour immunity [104], and the manufacturing complexity and regulatory challenges associated with EV-based therapeutics are considerable and may limit their widespread adoption [154,158].

It is important to emphasise that, to date, no EV-based therapy has entered clinical trials in MM. The current evidence relies exclusively on preclinical studies, including in vitro experiments and murine models, which may not faithfully recapitulate the complexity of the human bone marrow microenvironment [95,158]. Key knowledge gaps include the lack of understanding of EV biodistribution and clearance mechanisms in humans, the uncertain safety profile of chronic EV administration, the absence of standardised potency assays to evaluate EV-based products, and the limited knowledge of how patient-specific factors such as disease stage and prior therapies influence EV composition and function.

We acknowledge that current EV-based approaches in MM remain largely experimental [158]. The field would benefit from rigorous benchmarking of EV isolation and characterisation protocols to establish consensus standards [91], as well as systematic preclinical studies addressing safety, biodistribution, and off-target effects before clinical trials can be initiated [153]. A realistic assessment is needed of whether EV-based strategies offer clear advantages over existing therapeutic modalities, including monoclonal antibodies, CAR-T-cells, and bispecific T-cell engagers, which are already well-established in MM treatment algorithms [34,163,164,165,166].

The successful clinical implementation of EV-based therapies in MM will require the resolution of major translational challenges related to scalability, manufacturing standardisation, quality control, and regulatory approval [154,158]. Notably, the considerable investment required for large-scale EV production raises questions about cost-effectiveness and accessibility, particularly in the context of already expensive MM treatment regimens [34].

In summary, although EVs undeniably play a central role in MM pathobiology [104,158], their therapeutic exploitation faces substantial challenges that should not be underestimated. The most plausible near-term application may be as complementary biomarkers for disease monitoring rather than as transformative therapeutic platforms [154]. Future research should prioritise addressing fundamental technical and biological barriers before pursuing clinical translation, with particular attention to establishing robust evidence of safety and efficacy in appropriately designed preclinical models that adequately recapitulate human disease [90,153].

The therapeutic strategies targeting EVs in MM are summarized in Table 3.

## 6. Conclusions

MM remains a complex and largely incurable malignancy, driven by dynamic interactions between malignant plasma cells and the BM microenvironment. Increasing evidence highlights immune dysregulation—particularly the aberrant activation of immune checkpoint pathways—as a key mechanism underlying disease progression, immune escape, and resistance to therapy. In parallel, extracellular vesicles have emerged as critical regulators of MM pathobiology. By transferring proteins, immune checkpoint molecules, and regulatory RNAs, MM-EVs actively remodel both immune and stromal compartments, reinforcing a permissive and immunosuppressive microenvironment. These findings underscore the dual role of EVs as both drivers of disease progression and potential therapeutic tools. Future research should focus on integrating immune checkpoint targeting with EV-based strategies, including inhibition of vesicle-mediated signalling and the development of EV-based drug delivery systems. A deeper understanding of these interconnected mechanisms will be essential for identifying robust biomarkers and advancing precision medicine approaches, ultimately improving clinical outcomes and quality of life for patients with MM.

Nevertheless, despite the significant progress described, the clinical translation of EV-based approaches still needs to be fully addressed before their clinical implementation. Key challenges include the need for standardised and reproducible methods for EV isolation and characterisation, the development of scalable manufacturing processes suitable for clinical use, and a more comprehensive understanding of EV biodistribution and targeting in vivo. Additionally, robust clinical validation through well-designed trials is required to establish the safety, efficacy, and long-term impact of EV-based interventions. Addressing these aspects will be crucial to really confirm the clinical potential of EV-based strategies and their implementation in clinical practice, thereby facilitating the transition from bench to bedside.

## Figures and Tables

**Table 1:** Effects of MM-EVs on different immune cell populations.

Immune Cell Type	Effect of MM-EVs	Mechanisms Involved	References
CD8+ T-cells	T-cells exhaustion	PD-1, TIGIT, and LAG-3+ Y cells increase IFN-γ–producing CD8^+^ T-cells decrease	[109,124]
CD4+ T-cells	T-cells suppression	Upregulation of PD-1 and CTLA-4	[109]
NK cells	Modulation of NK cytotoxic response	EVs-associated MICA binding to NKG2D	[134]
Macrophages	Polarisation of M0 macrophages	Acquisition of M2-like phenotype Upregulation of PD-L1	[118,135]
Monocytes	Acquisition of an immune modulatory phenotype	Upregulation of IL-6 and MMP-9 secretion	[136]
M-MDSCs	Acquisition of an immunosuppressive phenotype	Upregulation of IFN-α, IFN-γ, TNF-α, and IL-6/JAK/STAT3 signalling	[124]

**Table 2:** Effects of MM-EVs on BMSCs.

Effect of MM-EVs in BMSCs	Mechanisms Involved	References
BMSCs adhesion	Enrichment in proteins involved in migration (MYH4, CD166, CD44)	[140]
BMSCs release of cytokines related to tumour growth and tumour survival	EGFR pathway activation Ape1/NF-kB pathway miRNA transfer	[96,123,144]
Transformation into CAFs	Transfer of MM-EV miR-146 and miR-21	[147]

**Table 3:** Therapeutic strategies targeting EVs in MM.

Therapeutic Strategy	Source/Target of EVs	Main Evidence in MM	Main Limitations
Inhibition of sEV biogenesis/release	MM cells	GW4869 reduced exosome release, attenuated bone disease, and enhanced bortezomib activity in preclinical MM models [153].	Limited specificity; possible interference with physiologic EV signalling [153].
Inhibition of sEV uptake/binding	MM cells and BM niche	Strategy to block EV-driven microenvironmental support and resistance circuits [153,154].	Still largely preclinical; molecular targets not fully standardised [154]
Native or engineered sEVs as drug carriers	Donor non-tumour cells or engineered producer cells	Considered a promising nanovector platform for anti-MM compounds and RNA therapeutics [154,159]	Manufacturing, loading efficiency, biodistribution, and batch reproducibility [154]
Engineered immunotherapeutic exosomes	CAR-T-cells or other immune cells	CAR-T-derived exosomes showed potent antitumour activity in preclinical studies and are conceptually relevant to MM immunotherapy [155,156]	Evidence in MM remains mostly indirect or early-stage; no established clinical application yet [155,157]
EV-based antitumour vaccines	Tumour-derived or engineered EVs	Discussed as an emerging immunotherapeutic strategy in MM [154]	Risk of immunosuppressive cargo; need for careful engineering and validation [154]

## Data Availability

No new data were created or analysed in this study. Data sharing is not applicable to this article.

## Abbreviations

AKT	Protein Kinase B (PKB)
APCs	Antigen-Presenting Cells
Ape1	Apurinic/apyrimidinic endonuclease
Arg-1	Arginase 1
ASCT	Autologous Stem Cell Transplantation
B2M	Serum beta-2 microglobulin
BCMA	B-cell Maturation Antigen
BM	Bone Marrow
BMPC	Bone Marrow Plasma Cells
BMSCs	Bone marrow mesenchymal Stem Cells
BTLA B and T	Lymphocyte Attenuator
CAFs	Carcinoma-Associated Fibroblasts
CAR T	Chimeric Antigen Receptor T-cell
CD47	Cluster of Differentiation 47
cGAS-STING	Cyclic GMP-AMP synthase—stimulator of interferon genes
cMyc	Cellular Myelocytomatosis
CRAB	hyperCalcemia, Renal impairment, Anaemia, and Bone lesions
CTLA-4	Cytotoxic T-Lymphocyte Antigen 4
CTLs	Cytotoxic T Lymphocytes
CXCL1	C-X-C motif ligand 1
DCs	Dendritic cells
E2F	Early Region 2 binding Factor
EGFR	Epidermal Growth Factor Receptor
EVs	Extracellular vesicles
FFAR1	Free Fatty Acid Receptor 1
FIH-1	Factor Inhibiting HIF-1
FISH	Fluorescence in situ hybridization
FLC	Serum Free Light chain
HD	Healthy Donors
HIST1H2BG	Histone Cluster 1 H2B Family Member G
HLA	Human Leukocyte Antigen
HMGB1	High mobility group box 1
ICI	Immune Checkpoint Inhibitor
IFN-α	Interferon α
IFN-γ	Interferon γ
IGF1	Insulin-like Growth Factor 1
IL-10	Interleukin-10
IL-2	Interleukin-2
IL-6	Interleukin-6
IL-8	Interleukin-8
IMWG	The International Myeloma Working Group
ISS	International Staging System
ITIH2	Inter-Alpha-Trypsin Inhibitor Heavy Chain H2
JAK	anus Kinase
LAG-3	Lymphocyte-activation gene 3
LncRNA	Long non-coding RNA
M-MDSCs	Monocytic Myeloid-Derived Suppressor Cells
MAPK	Mitogen-Activated Protein Kinase
MASS	Mayo Additive Staging System
MDSCs	Myeloid-Derived Suppressor Cells
MGUS	Monoclonal gammopathy of undetermined significance
MHC	Major Histocompatibility Complex
MICA	MHC class I chain-related protein A
MICB	MHC class I chain-related protein B
MiRNA	microRNA
MM	Multiple Myeloma
MM-EVs	Multiple Myeloma-Derived Extracellular Vesicles
MMP9	Matrix Metallopeptidase 9
mTOR	Mammalian Target of Rapamycin
NF-kB	Nuclear Factor kappa-light-chain-enhancer of activated B cell
NK	Natural Killer
NKG2D	Natural Killer Group 2-member D
NKG2DL	Natural Killer Group 2-member D Ligand
ORR	overall response rate
PCs	Plasma Cells
PD-1	Programmed Death-1
PD-L1	Programmed Death-Ligand 1
PD-L2	Programmed Death-Ligand 2
PET/CT	positron emission tomography
PFS	progression-free survival
PI3K	Phosphatidylinositol 3-kinase
ps-EVs	Patient-derived plasma EVs
R-ISS	Revised International Staging System
RAS	Rat sarcoma
RRMM	Relapsed/Refractory Multiple Myeloma patients
S1P	Sphingosine-1-phosphate
SMM	Smouldering Multiple Myeloma
SPHK1	Sphingosine Kinase 1
STAT3	Signal Transducer and Activator of Transcription 3
TCRs	T-cell receptors
TGF-β	Tumour Growth Factor- beta
TIGIT	T-cell immunoreceptor with Ig and ITIM domains
TIM-3	T-cell immunoglobulin and mucin domain-containing three
TNF-α	Tumour Necrosis Factor-α
Tregs	Regulatory T-cells
ULBP1-6	UL16-Binding Protein 1-6
VEGF	Vascular Endothelial Growth Factor
VISTA	V-domain Ig suppressor of T-cell activation
WBLDCT	whole-body low-dose computed tomography
